# Molecular and Pharmacological Modulation of CALHM1 Promote Neuroprotection against Oxygen and Glucose Deprivation in a Model of Hippocampal Slices

**DOI:** 10.3390/cells9030664

**Published:** 2020-03-09

**Authors:** Javier Garrosa, Iñigo Paredes, Philippe Marambaud, Manuela G. López, María F. Cano-Abad

**Affiliations:** 1 Departamento de Farmacología, Facultad de Medicina, Universidad Autónoma de Madrid, 28029 Madrid, Spain; javier.garrosa@uam.es (J.G.); iparedes003@gmail.com (I.P.); manuela.garcia@uam.es (M.G.L.); 2 Instituto Teófilo Hernando, Facultad de Medicina, Universidad Autónoma de Madrid, 28029 Madrid, Spain; 3 The Feinstein Institutes for Medical Research, Northwell Health, Manhasset, NY 11030, USA; pmaramba@northwell.edu; 4 Instituto de Investigaciones Biomédicas del Hospital Universitario de la Princesa, Diego de León 62, 28006 Madrid, Spain

**Keywords:** CALHM1, Calcium, CGP37157, ischemia, neuroprotection

## Abstract

Calcium homeostasis modulator 1 (CALHM1) is a calcium channel involved in the regulation of cytosolic Ca^2+^ levels. From a physiological point of view, the open state of CALHM1 depends not only on voltage but also on the extracellular concentration of calcium ([Ca^2+^]) ions. At low [Ca^2+^]_e_ or depolarization, the channel is opened, allowing Ca^2+^ influx; however, high extracellular [Ca^2+^]_e_ or hyperpolarization promote its resting state. The unique Ca^2+^ permeation of CALHM1 relates to the molecular events that take place in brain ischemia, such as depolarization and extracellular changes in [Ca^2+^]_e_, particularly during the reperfusion phase after the ischemic insult. In this study, we attempted to understand its role in an in vitro model of ischemia, namely oxygen and glucose deprivation, followed by reoxygenation (OGD/Reox). To this end, hippocampal slices from wild-type *Calhm1*^+/+^, *Calhm1^+/−^*, and *Calhm1^−/−^* mice were subjected to OGD/Reox. Our results point out to a neuroprotective effect when CALHM1 is partially or totally absent. Pharmacological manipulation of CALHM1 with CGP37157 reduced cell death in *Calhm1*^+/+^ slices but not in that of *Calhm1^−/−^* mice after exposure to the OGD/Reox protocol. This ionic protection was also verified by measuring reactive oxygen species production upon OGD/Reox in *Calhm1*^+/+^ and *Calhm1^−/−^* mice, resulting in a downregulation of ROS production in *Calhm1^−/−^* hippocampal slices and increased expression of HIF-1α. Taken together, we can conclude that genetic or pharmacological inhibition of CALHM1 results in a neuroprotective effect against ischemia, due to an attenuation of the neuronal calcium overload and downregulation of oxygen reactive species production.

## 1. Introduction

Ischemic stroke is the most frequent cause of cerebrovascular disease [1]. It is the third leading cause of death and the main cause of adult disability worldwide. Despite this high unmet medical need, there is no current effective neuroprotective treatment [2]. To date, thrombolytic or mechanical removal of the occlusion are the only therapeutic options. Mechanical thrombectomy complications occur in 11% of the patients and the intravenous thrombolytic treatment, using recombinant tissue plasminogen activator (rt-PA), has limitations [3,4,5]. During ischemic stroke, the acute occlusion of a vessel produces a rapid central core of brain infarct tissue where cells suffer necrosis. This core area is surrounded by a hypoxic but potentially salvageable tissue—the ischemic penumbra, where the blood flow reduction is not so drastic [6].

The drop in blood flow triggers a decrease of oxygen and glucose supply in the infarct area, promoting a disruption in the electron transport chain that leads to mitochondrial failure and a reduction of ATP levels. This decrease in ATP induces the malfunctioning of different membrane pumps such as Na^+^/K^+^/ATPase and Ca^2+^/ATPase, promoting membrane depolarization due to Na^+^ influx. This depolarization provokes the opening of voltage-dependent calcium channels (VDCCs) and makes the Na^+^/Ca^2+^ exchanger work in its reverse way; these two processes lead to intracellular Ca^2+^ overload [7,8]. This massive entry of Ca^2+^ into the neurons induces glutamate excitotoxicity, triggers the production of reactive oxygen species (ROS), and the release of inflammatory cytokines that ultimately lead to neuronal death [9,10,11,12]. Therefore, maintenance of intracellular Ca^2+^ homeostasis is crucial for cellular survival and function [11]. 

Considering the deleterious effect of Ca^2+^ dysregulation during ischemia, and the lack of efficacy of voltage-dependent calcium channel inhibitors in stroke clinical trials [13], finding new molecular targets that could be involved in modulating Ca^2+^ concentration in the neuron could provide a novel neuroprotective strategy for brain ischemia conditions. *Calhm1* is a gene discovered in 2008 in search of human genes with enriched expression in the hippocampus, which has been linked to enhanced risk for late-onset of Alzheimer’s disease (AD). This gene codifies a plasma membrane glycoprotein called CALHM1, which allows Ca^2+^ influx into the cells. Due to its high permeability to Ca^2+^, this ion channel turns out to be very important in cytosolic Ca^2+^ homeostasis regulation. CALHM1 ion channel is widely located in the central nervous system (CNS), mainly in neurons in both plasma membrane and endoplasmic reticulum (ER) [14].

CALHM1 is an octamer whose monomers form the functional ion channel [15]. CALHM1 opening is regulated by membrane potential and extracellular Ca^2+^ concentration ([Ca^2+^]_o_). In the presence of physiological [Ca^2+^]_o_ (~1.5 mM), CALHM1 is closed at resting membrane potentials but can be activated by strong depolarizations. On the contrary, hyperpolarization can trigger its inactivation, which prevents Ca^2+^ entry into the cell [16,17]. Furthermore, removal of calcium from the extracellular medium and subsequent Ca^2+^ add-back strongly elevates [Ca^2+^]_i_ in CALHM1-transfected HT-22 and N2A cells [14]. Finally, [Ca^2+^]_o_ oscillations can also regulate the CALHM1 state.

Related to its physiological roles, CALHM1 contributes to enhanced neuronal excitability in response to low [Ca^2+^]_o_. Reducing [Ca^2+^]_o_ from 1.5 to 0.2 mM reduces the input resistance by ~50% and increases excitability in neurons from wild-type mice but fails to enhance the excitability of cortical neurons from *Calhm1^−/−^* mice [17]. Also, CALHM1 participates in taste perception, acting as an ATP-releasing channel from type 2 taste bud cells [18].

CALHM1 is implicated in the Long-Term Potentiation (LTP) process, the molecular mechanism involved in learning and memory formation in the brain. Its opening triggers an increase in the phosphorylation of AMPA and NMDA receptors, promoting its trafficking to the plasma membrane, making them functional [19].

Lastly, CALHM1 has been implicated in brain ischemia. In *Calhm1*^−/−^ mice, the infarct volume after middle cerebral artery occlusion (MCAO) is significantly lower compared to wild-type mice. Also, its pharmacological blockade with Ruthenium Red (RuRed) or its silencing by using short hairpin RNA specific for CALHM1 (sh-CALHM1) induces neuroprotection in cultured cortical neurons subjected to oxygen and glucose deprivation (OGD) [20]. However, the molecular mechanisms underlying this neuroprotective process is still unknown.

The benzothiazepine CGP37157 was the first organic compound shown to have the ability to modulate the Ca^2+^ influx through CALHM1; at 1 μM, it reduces by over 50% the [Ca^2+^]_i_ after the removal and following the add-back of Ca^2+^ (conditions by which CALHM1 is activated) in Hela cells overexpressing CALHM1 [21]. Alternatively, CGP37157 is recognized as a blocker of the Na^+^/Ca^2+^ exchanger located in the mitochondrial membrane and partially blocks voltage-gated Na^+^ and L-type voltage-dependent calcium channels. This compound, which is able to cross the BBB, has been shown to exert neuroprotective properties in different oxidative stress models [22].

In this context, we aimed to identify by which molecular mechanisms the absence or blockade of CALMH1 exerts a protective effect under brain ischemia conditions by using hippocampal slices of *Calhm1*^+/+^, *Calhm1*^+/−^, and *Calhm1*^−/−^ mice.

## 2. Materials and Methods

### 2.1. Animal Usage and Care 

Native male and female mice (C57BL/6J) for CALHM1, *Calhm1^+/−^* and *Calhm1^−/−^* were used in the experiments following The Guide for the Care and Use of Laboratory Animals and were previously approved by the Institutional Ethics Committee of the Universidad Autónoma de Madrid, Spain. Mice were housed under controlled lighting and temperature conditions, and water and food were administered ad libitum. All efforts were made to minimize the number of animals used and their suffering. 

### 2.2. Isolation and Preparation of Mouse Hippocampal Slices

Adult mice (2–4 months) were used to obtain hippocampal slices (HSs). Mice were quickly decapitated by cervical dislocation and brains were removed from the skull and placed into cold Krebs bicarbonate dissection buffer (pH 7.4), containing: KCl 2 mM, NaCl 120 mM, NaHCO_3_ 26 mM, CaCl_2_ 0.5 mM, KH_2_PO_4_ 1.18 mM, MgSO_4_ 10 mM, sucrose 200 mM, and glucose 11 mM. The hippocampus was placed in a McIlwain tissue chopper and was cut up in 300-µm-thick hippocampal slices. After that, slices were introduced into a chamber at 34 °C and exposed to 95% O_2_/5% CO_2_ mixture for 45 min stabilization period before the beginning of the ischemic procedure.

### 2.3. Oxygen and Glucose Deprivation in Mouse Hippocampal Slices

In order to perform the ischemic protocol, one group of hippocampal slices were incubated in a Krebs solution named as control, composed by: NaCl 120 mM, NaHCO_3_ 26 mM, MgSO_4_ 1.19 mM, KCl 2 mM, KH_2_PO_4_ 1.18 mM, CaCl_2_ 2 mM, and glucose 11 mM. This mixture was bubbled with 95% O_2_/5% CO_2_. On the other hand, another pool of slices were subjected to oxygen-glucose deprivation (OGD) for 15 min. For this, slices were incubated in a glucose-free Krebs solution, where glucose was replaced by 2-deoxyglucose and equilibrated with a 95% N_2_/5% CO_2_ gas mixture. Thereafter, the OGD solution was removed, and oxygenated Krebs solution with glucose (reoxygenation period) was added and maintained for 2 h. When CGP37157 (10 and 30 μM) was used, it was added during the OGD and Reox periods. 

### 2.4. Glutamate Excitotoxicity in Mouse Hippocampal Slices

Apart from hypoxia, Ca^2+^ overload-induced by excessive glutamate release is one of the most important molecular events that takes place during an ischemic insult. With the aim of mimicking this process in vitro, we subjected the slices to an excitotoxicity protocol well described in the literature [23]. First, the hippocampal slices were subjected to a stabilization period in a solution composed of MgSO_4_ 1.19 mM, CaCl_2_ 2 mM, NaCl 120 mM, KH_2_PO_4_ 1.18 mM, KCl 2 mM, glucose 11 mM and NaHCO_3_ 26 mM, with 95% O_2_/5% CO_2_. After that, we immersed the slices in a culture media containing: 50% of Dulbecco’s Modified Eagle’s Medium (DMEM), 50% of Krebs–Ringer Bicarbonate Buffer, 100 units/mL penicillin and 100 mg/mL streptomycin, HEPES 20 mM and 1 mM glutamate for 4 h in a CO_2_ atmosphere at 37 °C. CGP37157 (10 μM) was applied as a CALHM1 antagonist and co-incubated for 4 h in the presence of glutamate.

### 2.5. Determination of Hippocampal Viability by the MTT Assay

Cell viability was measured by adding the MTT (3-(4,5-dimethylthiazol-2-yl)-2,5-diphenyltetrazolium bromide) to the hippocampal slices. This method is based on the ability of functional mitochondrial dehydrogenases to reduce the tetrazolium ring of MTT in an insoluble salt called formazan; being the amount of salt precipitate an indicator of cell viability. After incubation of hippocampal slices for 30 min in the presence of MTT (0.5 mg/mL) in Krebs solution at 37 °C, the formazan salt was solubilized with 200 μL of dimethyl sulfoxide (DMSO) for another 30 min, resulting in a purple-colored substrate. Finally, we measured the absorbance at 540 nm wavelength in an ELISA microplate reader. Absorbance values of control slices from wild-type mice were considered as 100% viability.

### 2.6. Quantification of Reactive Oxygen Species Production

To determine the production of reactive oxygen species (ROS) in hippocampal slices, we used the fluorescent dye 2’,7-Dichlorofluorescein diacetate (H_2_DCFDA) purchased from Molecular Probes (Invitrogen, Madrid, Spain). This compound crosses the plasma membrane and is cleaved by cytosolic esterases from the non-fluorescent product dichlorodihydrofluorescein, which binds to intracellular H_2_O_2_ generating dichlorofluorescein (DCF), a green fluorescent probe [24].

During the reoxygenation period, slices were loaded for 45 min with 10 μM H_2_DCFDA in Krebs solution and to stain nuclei, Hoechst33342 at 1 μg/mL was added for 5 min. Then, hippocampal slices were washed with the control Krebs solution. The fluorescence records were determined in a fluorescence inverted NIKON eclipse T2000-U microscope. The excitation and emission wavelengths were 485 and 520 nm, respectively and analysis was performed using the Metamorph program version 7.0

The area of interest was the CA1 region of the hippocampus, and images were obtained at magnifications of 10×. ROS production was calculated as the ratio of the mean DCFDA intensity divided by the mean Hoechst fluorescence. Data from control slices of wild-type were taken as 100%. 

### 2.7. Western Blot Analysis

Slices of the different experimental groups were lysed in 100 μL cold lysis buffer (1% Nonidet P-40, 1 mM PMSF, 20 mM Tris-HCl pH 7.5, 10% glycerol, 20 mM NaF, 137 mM NaCl, 1 μg·mL−1 leupeptin, 1 mM Na_3_VO_4_ and 1 mM sodium pyrophosphate) and disaggregated with Sonicator Ultrasound. The resulting tissue mixture was centrifuged at 13,000 rpm for 5 min at 4 °C, and the supernatant was collected. From each supernatant, 30 μg of protein were resolved by SDS-PAGE and transferred to Immobilon-P membranes (Millipore Corp.GE). Membranes were incubated with anti-HIF-1α at 1:500 (Cayman Chemical, Michigan, MI, USA). Seahorse peroxidase-conjugated secondary antibodies (1:10,000) were used to detect proteins by chemiluminescence. Protein bands density was analyzed using the ImageJ analysis software (National Institutes of Health, USA).

### 2.8. Statistical Analysis

Results are represented in bars as mean ± S.E.M. Statistical differences were determined by one-way ANOVA test followed by Newman Keuls post hoc and paired t-test. The program used for the analysis was GraphPad Prism 5.00. The threshold of statistical significance was established at *p* < 0.05.

## 3. Results

### 3.1. Partial or Total Molecular Ablation of CALHM1 Results in a Protective Effect in Hippocampal Slices Exposed to OGD/Reox

**Figure 1:**
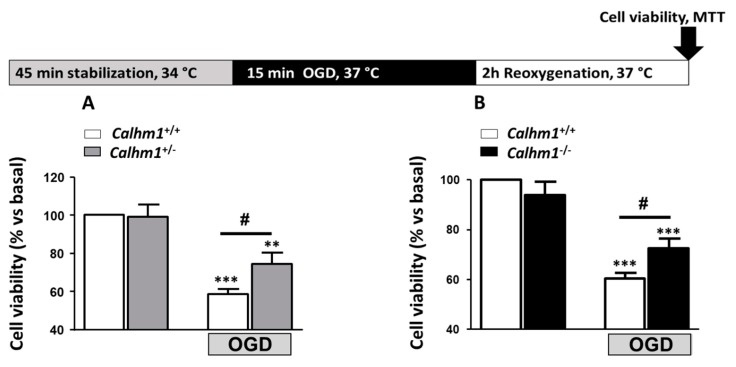
*Calhm1^−/−^* mice present neuroprotection against cell death elicited by oxygen and glucose deprivation followed by reoxygenation (OGD/Reox) in hippocampal slices. The top part of the figure shows a schematic representation of the OGD/Reox protocol used, as described in materials and methods. (**A**) Represents cell viability measured by MTT reduction in hippocampal slices from *Calhm1*^+/+^ and *Calhm1^+/−^* mice under basal conditions or exposed to the OGD/Reox protocol. OGD/Reox-induced cytotoxicity is prevented in *Calhm1^+/−^* hippocampal slices. Data are the mean ± S.E.M of seven different animals; all variables were run in parallel. ^***^ *p* < 0.001 and ^**^ *p* < 0.01 in comparison to basal; ^#^ *p* < 0.05 respect to *Calhm1*^+/+^ OGD. (**B**) Hippocampal slices from *Calhm1^−/−^* mice subjected to OGD/Reox show higher cell viability when compared to those obtained from *Calhm1*^+/+^ mice. Data represent the mean ± S.E.M of 7 different animals; all variables were run in parallel. ^***^ *p* < 0.001 versus basal; ^#^ *p* < 0.05 respect to *Calhm1*^+/+^ OGD. One-way ANOVA followed by Newman–Keuls was performed. Data were normalized to *Calhm1*^+/+^, which was considered as 100% of viability.

Hippocampal slices from *Calhm1*^+/+^, *Calhm1^+/−^* and *Calhm1^−/−^* mice were subjected to the OGD/Reox protocol previously described. Considering the high Ca^2+^ permeability of CALHM1, and the excessive release of this cation to the synaptic cleft during an ischemic insult, it is presumable that the lack of the channel exerts a beneficial effect against ischemic damage. The OGD/Reox protocol was carried out and cell viability was determined by the MTT method in hippocampal slices from each group. Figure 1A, illustrates the cell viability of hippocampal slices from *Calhm1*^+/+^ and *Calhm1^+/−^* mice. Slices from animals with deletion of one allele of CALHM1 showed significantly higher viability (74.2% ± 6.2) compared to wild-type (58.4% ± 2.7) when subjected to OGD/Reox condition. Following the same experimental approach, we also observed that slices from animals with total ablation of *Calhm1^−/−^* showed less toxicity in comparison to those from *Calhm1*^+/+^ mice (60.4% ± 2.3 vs. 72.5% ± 4, respectively) (Figure 1B). These results indicate that either partial or total genetic deletion of CALHM1 exerts a neuroprotective effect against OGD/Reox damage in our ex-vivo model of brain ischemia. 

### 3.2. Genetic Ablation of CALHM1 Promotes Neuroprotection in the Glutamate Excitotoxicity Model

Due to the fact that the absence of CALHM1 proved to be neuroprotective in the OGD/Reox model, new experiments were performed, but this time focusing on the neurotransmitter glutamate, known to be cytotoxic during brain ischemia. As previously described, glutamate induces neurotoxic effects due to activation of NMDA and metabotropic receptors, leading to massive Ca^2+^ entry and cell death.

Exposure of *Calhm1*^+/+^ hippocampal slices to 1 mM glutamate during 4 h, reduced cell viability to 59.9% ± 3.8; however, slices from *Calhm1^−/−^* were significantly protected (70.5% ± 6). 

**Figure 2:**
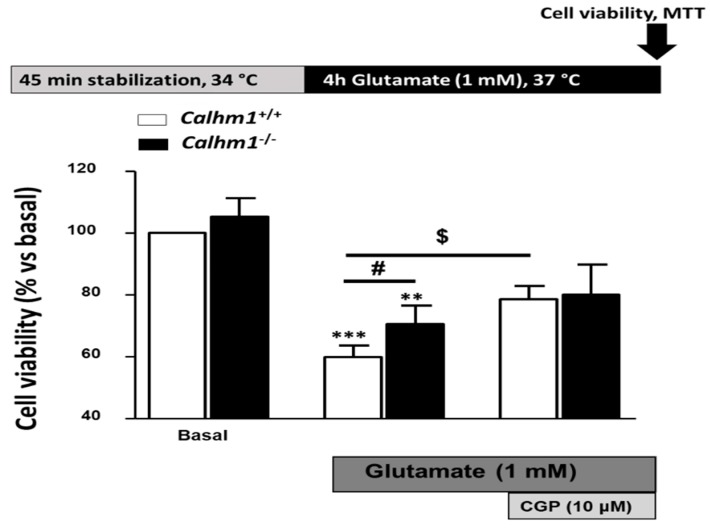
CALHM1 ablation results beneficial against glutamate-induced excitotoxicity and abolishes the neuroprotective properties of CGP37157. The top part of the figure illustrates a schematic representation of the glutamate induced excitotoxicity protocol. Excitotoxicity mediated by glutamate (1 mM, 4 h) in hippocampal slices was measured by the MTT method. As represented, slices from *Calhm1^−/−^* mice showed significantly less cell damage than *Calhm1*^+/+^ slices. Moreover, the neuroprotective profile described previously for CGP37157 (10 μM) in the glutamate model of ischemia was partially lost when CALHM1 was not present. Data correspond to the mean ± S.E.M of five different mice; all conditions were performed in parallel. ^***^ *p* < 0.001 and ^**^ *p* < 0.01 compared to basal; ^#^ *p* < 0.05 versus *Calhm1*^+/+^ glutamate; ^$^ *p* < 0.05 respect to *Calhm1*^+/+^ glutamate. Paired T-Test was performed. Data were normalized to *Calhm1*^+/+^, considering it as 100% of cell viability.

As previously described in our laboratory [21], CGP37157 reduces Ca^2+^ influx through CALHM1 in Hela cells. Hela cells lack L-type VDCCs, and when transiently transfected with CALHM1, the only Ca^2+^-permeant channel expressed in the plasma membrane is CALHM1. So, according to our previous results, CGP37157 blocks CALHM1 since in control Hela cells, the contribution of the Na^+^/Ca^2+^ exchanger upon Ca^2+^ add-back protocol seems to be insignificant compared with the Ca^2+^ entry in those cells overexpressing CALHM1. Therefore, we carried out experiments to determine the protective role of this compound in our models. The addition of the CGP37157 (10 μM) to the medium during the glutamate incubation period increased cell viability significantly in hippocampal slices from *Calhm1^+^*^/+^ mice, but it did not provide significant protection in *Calhm1^−/−^* slices. The increase in cell survival in *Calhm1^+^*^/+^ slices may be related to the blocking action on NMDAR, VDCCs and sodium calcium exchanger (NCX) reported for CGP37157, preventing the Ca^2+^ overload upon exposure to high glutamate concentrations [22]. This effect is higher in *Calhm1^+/+^* slices because CGP37157 is also blocking Ca^2+^ influx through CALHM1, which is not present in *Calhm1^−/−^* slices (Figure 2). These findings highlight the idea that in hippocampal slices from *Calhm1^−/−^* mice, CGP37157 is not exerting neuroprotection mediated by blockade of CALMH1 channels. Taken together, these results support that the genetic deletion or the pharmacological blockade of CALHM1 attenuates the massive release of glutamate and Ca^2+^ overload to provide neuroprotection. However, the effects of CGP37157 on voltage dependent calcium channels (VDCCs) in CALHM1 mouse models have still not been reported, but further investigations are required to solve this crucial point.

### 3.3. CGP37157 Requires CALHM1 to Exert Neuroprotection under OGD/Reox Conditions 

**Figure 3:**
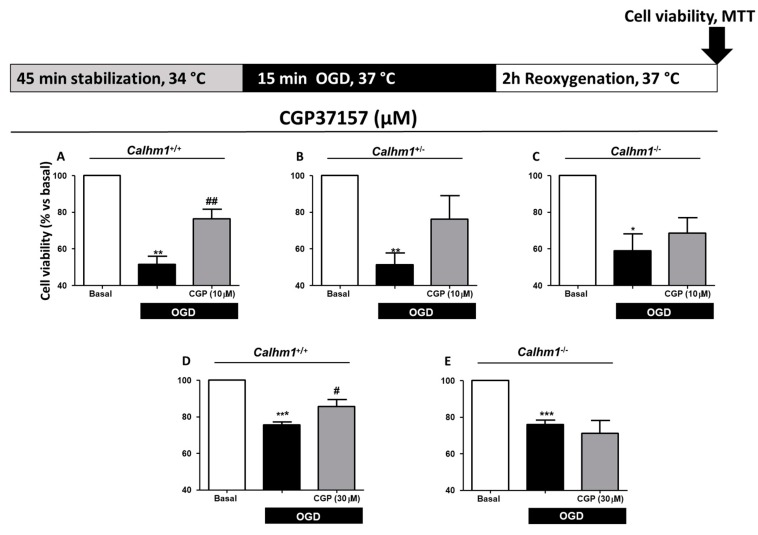
CALHM1 is required for the protective effect of CGP37157 in the model of oxygen and glucose deprivation followed by reoxygenation (OGD/Reox). Representation of the OGD/Reox protocol used on the top of figures A-E. (**A**–**C**) Cell viability in hippocampal slices of *Calhm1*^+/+^, *Calhm1^+/−^* and *Calhm1^−/−^* mice was determined through the MTT method. Treatment with CGP37157 (10 μM) during the OGD/Reox protocol only provided neuroprotection in *Calhm1*^+/+^ slices (**A**), while this effect was not observed in *Calhm1^+/−^* (**B**) or *Calhm1^−/−^* (**C**) genotypes. (**D**–**E**) CGP37157 (30 μM) only exerted benefits in *Calhm1*^+/+^ slices (**D**), without having an effect on *Calhm1^−/−^* samples (**E**). Data represent the mean ± S.E.M of 3–5 different animals; all variables were run in parallel. ^***^ *p* < 0.001, ^**^ *p* < 0.01 and ^*^ *p* < 0.05 compared to basal; ^##^ *p* < 0.01 and ^#^ *p* < 0.05 respect to OGD. One-way ANOVA followed by Newman–Keuls was performed. Data were normalized to the basal condition which was considered as 100% of viability.

As mentioned above, previous findings from our laboratory [21] demonstrated that CGP37157 modulated massive Ca^2+^ entry through CALHM1 by blocking it. Due to the properties of CGP37157, new experiments were performed in *Calhm1^+^*^/+^, *Calhm1^+/−^*, and *Calhm1^−/−^* mice hippocampal slices exposed to OGD/Reox in the presence or absence of CGP37157 at 10 μM. The OGD/Reox protocol induced 51.9% ± 4.3 cell death in *Calhm1^+^*^/+^ hippocampal slices, while the incubation of CGP37157 (10 μM) reverted the cytotoxic effect to 76.3% ± 5.3 (Figure 3A). Incubation with 10 μM of CGP37157 of *Calhm1^+/−^* or *Calhm1^−/−^* hippocampal slices *per se* did not induce an observable neuroprotective effect. Despite not observing a significant effect, cell viability was increased in the presence of CGP37157 (10 μM) compared to non-treated hippocampal slices of *Calhm1^+/−^* and *Calhm1^−/−^* mice (Figs. 3B and 3C). This increase in cell survival could be attributed to the blocking action of CGP37157 on NMDAR, VDCCs, and NCX, preventing the Ca^2+^ overload upon exposure to ischemic conditions [22]. Moreover, CGP37157 (30 μM) was neuroprotective in the OGD/Reox protocol in *Calhm1^+^*^/+^ slices (Figure 3D), but not in *Calhm1^−/−^* mice (Figure 3E). These results indicate that the pharmacological modulation of CALHM1 could be a novel neuroprotective mechanism under ischemic brain conditions.

### 3.4. The Production of Free Radicals Induced by OGD/Reox is Prevented when CALHM1 Is Absent

**Figure 4:**
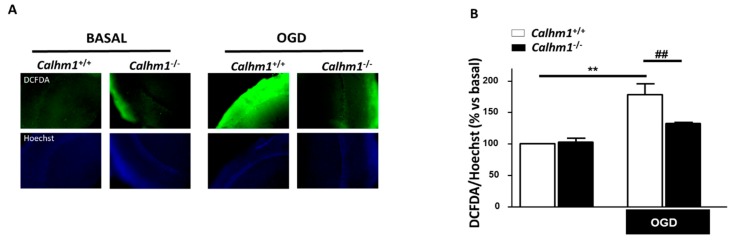
ROS production is attenuated in *Calhm1^−/−^* hippocampal slices upon OGD/Reox. (**A**) Images on the top show DCFDA fluorescence for ROS production and images on the bottom show Hoechst fluorescence as an indicator of nuclei density in the CA1 region of hippocampal slices from *Calhm1*^+/+^ and *Calhm1^−/−^* mice, under basal or after OGD/Reox exposure. (**B**) Quantitative analysis of ROS production is indicated as the ratio of DCFDA/Hoechst fluorescence intensity. Bars represent the means ± S.E.M of three different animals from each group; all variables were performed in parallel. ** *p* < 0.01 versus basal *Calhm1*^+/+^; ^##^ *p* < 0.01 compared to OGD *Calhm1*^+/+^. One-way ANOVA followed by Newman-Keuls was made. Data were normalized to *Calhm1*^+/+^, which was considered as 100% of viability.

It is well described that under hypoxic conditions, such as ischemia, there is intracellular Ca^2+^ overload and ROS production. Those pathological conditions induce overactivation of astrocytes and microglia. These two cell types in brain parenchyma under pathological situations lead to an increase in the production and release of ROS and inflammatory mediators, worsening neuronal damage [25]. Once demonstrated that CALHM1 ablation has a neuroprotective profile, we decided to go further by measuring ROS production in hippocampal slices from each experimental group. Figure 4A illustrates the production of ROS in the CA1 region of hippocampal slices of both, *Calhm1*^+/+^ and *Calhm1^−/−^* mice measured by the fluorescent probe DCFDA (green). In basal conditions *Calhm1*^+/+^ and *Calhm1^−/−^* showed similar ROS release as represented in the Figure 4B; however, after exposure to OGD/Reox, slices from *Calhm1*^+/+^ mice showed significantly higher levels of free radical production in comparison with those from *Calhm1^−/−^* (178.1% ± 17.01 vs. 132.6% ± 1.97, respectively). These findings support the idea that the absence of CALHM1 not only decreases ^[Ca2+^]_c_ entry but, in addition, contributes to lowering ROS production, opening a new neuroprotective pathway for CALHM1.

### 3.5. HIF-1α is Overexpressed under OGD/Reox Conditions when CALHM1 is Absent

**Figure 5:**
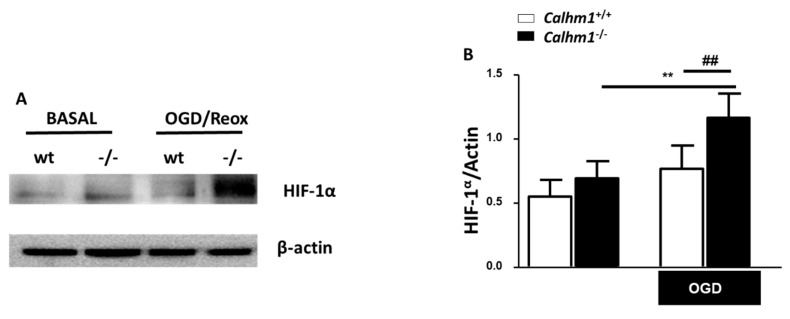
HIF-1α protein is upregulated in *Calhm1^−/−^* hippocampal slices after exposure to OGD/Reox. (**A**) Representative image of the bands showing the expression of HIF-1α by western-blotting obtained from *Calhm1*^+/+^ and *Calhm1^−/−^* hippocampal slices subjected to OGD/Reox. (**B**) Quantitative expression of HIF-1α is represented in bars using β-actin for normalization. Data are the mean ± S.E.M of eight different animals from each group. ** *p* < 0.01 versus basal *Calhm1^−/−^*; ## *p* < 0.01 compared to OGD *Calhm1*^+/+^. Paired t-test was performed.

To further understand the molecular mechanism by which the lack of CALHM1 induces neuroprotection upon OGD/Reox conditions, new experiments were carried out, but this time we searched for a Ca^2+^ dependent protein such as HIF-1α. Under hypoxic conditions, HIF-1α accumulates and dimerizes with HIF-1β, and translocates into the nucleus where it binds to the hypoxic-response elements (HREs), inducing the expression of genes that have been linked with the adaptation to hypoxia, such as antioxidant genes, as well as genes involved in angiogenesis and glycolytic energy metabolism. This metabolic adaptation decreases the formation of ROS and mitochondrial toxicity [26]. As illustrated in Figure 4, *Calhm1^−/−^* slices showed a reduction in the production of free radicals, in consequence, some antioxidant or pro-survival pathways could be activated. Due to its role as a master regulator in hypoxia, we decided to study HIF-1α levels in *Calhm1*^+/+^ and *Calhm1^−/−^* mice. As shown in Figure 5, HIF-1α expression was significantly higher in *Calhm1^−/−^* hippocampal slices upon OGD/Reox compared to *Calhm1*^+/+^ slices. 

## 4. Discussion

In the present work, we have shown that the partial or total absence of CALHM1 results neuroprotective against neuronal death in an ex vivo model of brain ischemia, mice hippocampal slices subjected to OGD/reox. We hypothesized that the Ca^2+^ channel CALHM1 could worsen cell damage upon ischemia-reperfusion due to its high Ca^2+^ permeability [17]. Recently, it has been established that the CALHM1 channel is implicated in ischemia brain damage [20]. These previous findings were focused on *Calhm1^−/−^* mice without explaining the effects of hypoxia in *Calhm1^+/−^* mice. In cortical neurons transfected with an shRNA-CALHM1 [20] showed a 13% reduction in lactate dehydrogenase release, indicating a mild contribution of CALHM1 to brain-ischemia neurotoxicity. Considering that presumably, CALHM1 is exerting its cytotoxic effect during the reperfusion phase of brain ischemia, due to its activation properties (Ca^2+^ add-back protocol), we hypothesize that the absence of one allele of CALHM1 should be sufficient to reduce a large amount of Ca^2+^ influx into the neurons during the reoxygenation period, exerting a similar neuroprotective effect to the total ablation of CALHM1 gene. Also, during a hypoxic insult in the brain, the depolarization wave and the excessive amount of glutamate released to the synaptic cleft, activates VDCCs and NMDAR, respectively, being the effects of these two types of channels predominant on CALHM1 participation [7,8,9,10]. As an example, heterozygous mice of the aryl hydrocarbon receptor (AhR*^+/−^* mice) show a significant reduction in infarct volume as well as an improvement of neurological deficits after a middle cerebral artery occlusion (MCAO) in comparison to wild-type mice [27]. These results indicate that the deletion of only one allele of AhR results in neuroprotectivity against brain ischemia, similar to our observations with *Calhm1^+/−^* mice. 

In view of the physiological role of CALHM1 in the regulation of AMPA and NMDA receptor expression and its implication in memory consolidation in the hippocampus [19], as well as, the participation of CALHM1 in the maintenance of neuronal excitability [17], we decided to perform our experiments in brain hippocampal slices from *Calhm1*^+/+^, *Calhm1^+/−^* and *Calhm1^−/−^* mice. In this study, we have performed experiments with *Calhm1^+/−^* mice, showing that the deletion of just one allele of CALHM1 results neuroprotective in the OGD/Reox protocol. In addition, we have seen that pharmacological treatment with CGP37157 resulted neuroprotective upon OGD/Reox conditions in *Calhm1*^+/+^ mice, whereas this effect was reduced in *Calhm1^+/−^* or *Calhm1^−/−^* hippocampal slices; however, protection elicited by CGP37157 in *Calhm1*^+/+^ mice did not reach basal condition values. These finding could be related to the reported lack of protective effects of CGP37157 against oxidative stress cell damage; for example, in SHSY5Y neuroblastoma cells subjected to oxidative stress stimulus such exposure to Oligomycin/Rotenone, CGP37157 was not neuroprotective [22]. OGD/Reox increases oxidative stress, therefore, under these conditions, it would be expected that CGP37157 would not be able to provide significant neuroprotection. These results indicate that the expression of CALHM1 seems to be necessary to induce pharmacological neuroprotection with CGP37157 (Figure 3). As mentioned earlier [21], it has been reported that CGP37157 can block Ca^2+^ flux through the mitochondrial sodium/calcium exchanger (mNCX), L-type VDCCs, the plasmalemmal sodium/calcium exchanger (pNCX), and voltage-gated sodium channels, being more selective at mNCX [28]. However, there is currently no experimental evidence in the literature that describes any alteration in the expression or functionality of mNCX, voltage-gated sodium channels, and L-type VDCC when CALHM1 is absent, so it should not affect the expression or function of these channels. 

CALHM1 controls [Ca^2+^]_c_ [15,16,17], mitochondrial [Ca^2+^] [29] and ER [Ca^2+^] [30]. Now we have more evidence that the opening of CALHM1 upon ischemia in the OGD/Reox model or glutamate excitotoxicity, induces large amounts of Ca^2+^ entry and plasma membrane depolarization [15], but its pharmacological blockade with CGP37157 reduced cell death in *Calhm1*^+/+^, but not in *Calhm1^+/−^* and *Calhm1^−/−^* mice. In other words, in hippocampal slices from *Calhm1*^+/+^ mice, OGD/Reox-induced cell death can be pharmacologically prevented by CGP37157 by a mechanism that implicates inhibition of CALMH1 channels. These results demonstrate that the Ca^2+^ driven through CALHM1 plays a neurotoxic effect and its blockade results neuroprotective. To further elucidate the neuroprotective properties of CALHM1, we performed experiments with *Calhm1^+/−^* mice. Upon OGD/Reox, the partial lack of CALHM1 exerts neuroprotective properties, pointing out that the possibility of only the deletion of one allele of CALHM1 is enough to induce neuroprotection against brain ischemia. However, in the model of glutamate toxicity, hippocampal slices from *Calhm1^+/−^* genotype did not show the cytoprotective effect present in those from *Calhm1^−/−^* mice (data not shown). To go further, we decided to investigate the molecular pathway by which the absence of CALHM1 resulted in neuroprotection. Mitochondria efficiently buffer Ca^2+^ driven through CALHM1 channels [29]. Ca^2+^ entering through the mitochondrial uniporter is translated to more hyperpolarized mitochondrial membrane potential and to more ATP production, but much more ROS production [31]. In fact, this was the case in our experimental conditions; *Calhm1*^+/+^ hippocampal slices exposed to OGD/Reox released higher amount of ROS than the *Calhm1^−/−^* ones. These findings highlight that the deletion of CALHM1 results neuroprotective not only to the reduction of Ca^2+^ entry driven by the channel but also to lower ROS production. Focusing on our results, CALHM1 absence results in high cell viability upon exposure of hippocampal slices to ischemia-related protocols and less ROS production in the CA1 hippocampal region.

Another key marker activated after exposure to OGD/Reox is HIF-1α. This protein is inducible upon hypoxia and cytosolic Ca^2+^ elevation, triggering cell survival under low oxygen concentrations [32]. We found a strong elevation of HIF-1α in the hippocampus of *Calhm1^−/−^* mice, subjected to OGD/Reox, compared to *Calhm1*^+/+^. An increase in HIF-1α levels might be beneficial because it could further induce antioxidant protein expression [33]. The finding that HIF-1α is upregulated in *Calhm1^−/−^* samples is conflicting. However, intracellular Ca^2+^ increase is not the only molecular mechanism that induces HIF-1α expression and stabilization. HIF-1α stabilization in the cytosol promotes metabolic adaptation to hypoxia, proliferation, survival, and resistance to apoptosis, although the lack of CALHM1 leads to less Ca^2+^ entry into the cell, it is no surprising that the expression levels of HIF-1α protein are increased in knock-out mice. Also, HIF-1α regulates the expression of some Ca^2+^ permeant channels, such as the transient receptor potential (TRP) C1 (TRPC1), stromal interaction molecule-1 (STIM1), or the Golgi Ca^2+^ pump secretory pathway Ca^2+^-ATPase 2 (SPCA2). Based on the latter observations, we can hypothesize that the deletion of CALHM1 could trigger an elevation in HIF-1α levels as a compensatory mechanism in order to restore Ca^2+^ signaling through other Ca^2+^ channels [34]

Although different CALHM1 antagonists have been described in the literature such as Ni^2+^ and Co^2+^, their high toxicity and non-specificity make them inadequate for use in the clinic. Something similar occurs with 2-APB and Ruthenium Red [17], they block CALMH1 channels but at high concentrations that could also be toxic. The organic properties of CGP37157 and its blocking action on CALMH1 channels at the micromolar range, contrary to other CALMH1 blockers previously described [17], indicate that it could be a druggable compound for stroke.

## 5. Conclusions

Genetic or pharmacological inhibition of CALHM1 in hippocampal slices from mice, was neuroprotective in the ODG/Reox protocol, suggesting that Ca^2+^ entry through CALHM1 contributes to cell death upon ischemic conditions. The neuroprotective molecular pathway results from lowering ROS production and activating of HIF-1α. Taken together, these results open a novel neuroprotective pathway for ischemic stroke, which includes the inhibition of CALMH1.
